# Cognitive Deficits among Individuals Admitted to a Post-Acute Pneumological Rehabilitation Unit in Southern Italy after COVID-19 Infection

**DOI:** 10.3390/brainsci13010084

**Published:** 2023-01-01

**Authors:** Gianvito Lagravinese, Giorgio Castellana, Fabio Castellana, Maddalena Genco, Rita Petrelli, Maria Ruccia, Maria Aliani, Mauro Carone, Rodolfo Sardone, Petronilla Battista

**Affiliations:** 1 Istituti Clinici Scientifici Maugeri SpA SB, IRCCS, Institute of Bari, 70100 Bari, Italy; 2 Unit of Research Methodology and Data Sciences for Population Health, National Institute of Gastroenterology “Saverio de Bellis”, Research Hospital, Castellana Grotte, 70013 Bari, Italy

**Keywords:** neuropsychological assessment, neurorehabilitation, SARS-CoV-2, COVID-19, cognitive disorders, respiratory medicine

## Abstract

(1) Background: We investigated the differences in the neuropsychological profile as well as the pneumological and motor functions in two groups of patients admitted to rehabilitation who received different respiratory support during their COVID-19 infection. (2) Methods: Group-1 (*n* = 18; 15 male, median age 67.5) consisted of patients who received non-invasive mechanical ventilation; Group-2 (*n* = 19; 16 male, median age 63) consisted of patients who received invasive mechanical ventilation. All patients underwent a neuropsychological assessment including Mini-Mental State Examination (MMSE), Frontal Assessment Battery (FAB), and the Repeatable Battery for the Assessment of Neuropsychological Status (R-BANS) to evaluate the patients’ cognition. Depression and anxiety were also measured at admission and discharge to rehabilitation. (3) Results: At admission, patients impaired at MMSE were 44% in Group-1 and 5% in Group-2, while patients impaired at FAB were 88% in Group-1 and 26% in Group-2. Wilcoxon’s effect size revealed meaningful differences between groups for FAB, R-BANS global score, immediate and delayed memory, and attention-coding task, with Group-2 performing better than Group-1 across all measures. At discharge, 52% of the 25 patients re-assessed still had mild to moderate cognitive deficits, while 19% had depression and 35% had anxiety. (4) Conclusions: Patients who received oxygen therapy experienced higher levels of acute and chronic stress compared to those who benefitted from invasive mechanical ventilation. Despite patients showing a meaningful improvement at discharge, cognitive impairment persisted in a great number of patients; therefore, long-term neuropsychological follow-up and treatment for COVID-19 patients are recommended.

## 1. Introduction

The coronavirus pandemic (COVID-19) is the most serious since the 1918 flu pandemic. Despite its pathophysiology being intensively studied in the past years, the cognitive effects of COVID-19 are not yet completely understood [1]. COVID-19 infection triggers a broad spectrum of symptoms; it is now recognized as a multi-organ disease where poor respiratory outcomes such as pneumonia, dyspnea, hypoxia, and reduced diffusion capacity [2] are not the only negative consequences of the disease. A wide range of symptoms can appear within four weeks after infection and may last for years, and become a known clinical condition called “post-COVID syndrome” or informally as “long-COVID” [3,4]. These lingering symptoms can be neurological (e.g., fatigue, muscular weakness, joint pain, myopathies, polyneuropathy, myalgia, headache, encephalitis, dysautonomia, vertigo, anosmia, and Guillain–Barre syndrome) and/or neuropsychiatric such as anxiety and depression [1,3,4,5,6,7].

Among the neurological sequelae, neuropsychological deficits (e.g., brain fog, dysexecutive syndrome) have been frequently reported [8,9,10]. The prevalence of cognitive impairment in patients who survived COVID-19 ranges from 2.6% [11] to 81% [12] at hospital discharge in an outpatient rehabilitation clinic setting. At 3 months after discharging home, patients evidencing cognitive impairment varied from 21% to 65% [13,14,15]. A follow-up study of 116 patients first admitted to the intensive care unit and then successfully discharged home found that survivors had an overall good functional and cognitive recovery at 2-months follow-up [11]. Although this study included a large sample of subjects and found encouraging follow-up outcomes, on the other hand, it lacked an extensive neuropsychological assessment as the investigation of cognition was limited to a telephone screening tool. More recently, Hadad et al. [16] found that mild to severe cognitive impairment persisted at 10–12 months after discharge home in 93% of patients visited for follow-up in an outpatient clinical setting, with 67% reporting decreased independence in IADLs (instrumental activities of daily living) due to their cognitive deficits. This study evaluated 46 patients using the Montreal Cognitive Assessment (MoCA) screening test and showed that patients were impaired in phonemic fluency and attention subtests, also demonstrating that cognitive decline may affect patients post-COVID, independently of disease severity. Overall, these studies employed a wide range of cognitive assessment tools, with the majority using screening tools that include few items and sometimes can overestimate the presence of cognitive deficits when items are too difficult, or underestimate the presence of cognitive deficits when items are too easy. The results concerning cognitive sequelae are determined by different types of samples and by the diverse design and settings of previous studies, most of which involved hospitalized patients within a cohort or cross-sectional designs. These and other methodological limitations of previous studies in the field were highlighted by Tavares-Junior et al. [15], who carried out a critical systematic review of cognitive impairment in confirmed COVID-19 patients and reported the wide variety of cognitive assessment instruments employed across studies influencing the outcome, being the majority of screening tools. Therefore, due to the variety of screening assessment tools used, it remains difficult to estimate the true prevalence of cognitive impairment at different stages (e.g., acute, subacute, and chronic) among patients who survived COVID-19.

Differences in cognition based on whether participants received invasive ventilation techniques have been also investigated. Overall, it is well-known that higher levels of respiratory distress are often associated with a higher risk of developing temporary or persistent cognitive impairment [17,18]. The pathological mechanisms that might underlie this phenomenon are related to the decreased alveolar oxygen exchange rate during inflammation, or edema causing cellular damage due to viral infections. Such mechanisms cause a chain of events including cerebral hypoxemia, vasodilatation, hyperemia, and neural swelling [18]. The consequences of changes in brain perfusion due to hypoxemia in patients with severe respiratory symptoms are associated with the presence of cognitive impairment [19]. The hippocampus, cingulate cortex, temporo-parieto-occipital cortices, and basal ganglia are among those brain regions more vulnerable to inflammation as they contain more enzymes involved in the inflammatory response [18]. These brain regions play an important role in memory, attention, and emotion processing [17,20,21]. Patients who experienced higher levels of respiratory distress may exhibit poorer performance on visual and verbal memory, processing speed, and social cognition tests [17,20,21]. The literature on COVID-19, however, is quite inconsistent, making it difficult to draw conclusions about whether the severity of COVID-19 differentially affects cognition, and whether patients require invasive ventilation. Some authors have suggested that cognitive deficits related to grade and chronicity of diminished oxygenation during illness may explain why deficits seem to be more accentuated in patients with severe illness compared to patients with mild-moderate illness. Previous studies found severe cognitive impairments in patients who received intensive care unit (ICU) care compared to those who received less intensive treatment [22,23]. Patients who are being treated within a critical care unit undergo prolonged isolation, weakness, and immobility and may develop acute organic brain syndrome. The ICU syndrome involves impaired intellectual functioning and can manifest a variety of psychological reactions including fear, anxiety, depression, hallucinations, and delirium, conferring a major determinant of poor long-term functional outcome [24]. In contrast, another study showed better cognitive performance in patients who underwent mechanical ventilation compared to non-mechanical ventilation, with the former group being significantly younger [25]. These results were also confirmed in a retrospective study on 152 patients that found better cognitive performance in patients who received ICU care compared to those who received oxygen therapy only [26], suggesting a protective role of the ICU on cognitive functions, as patients might suffer less from cerebral hypoxia compared to those treated with non-invasive ventilation. In an outpatient clinic, a report examined cognitive performance in 29 COVID-19 patients 3–4 months after their hospital discharge and found no correlation between the severity of COVID-19 infection, oxygen supplies needed, and cognitive impairments [14,27]. However, all of these studies employed brief cognitive screening tools to evaluate the neuropsychological profile of patients.

In light of the aforementioned literature, and on the basis that SARS-CoV-2 is considered neurotropic in humans [28,29,30], inducing a significant inflammatory response and affecting brain areas especially vulnerable to the action of inflammatory markers such as the hippocampus [18,21], we hypothesized that patients with COVID-19 demonstrate multiple cognitive impairments. Specifically, we investigated whether the type of respiratory assistance they benefited from during the acute phase of the disease had an impact on the cognitive outcomes. It is unclear whether persistent objective cognitive impairments after COVID-19 illness are related to higher levels of respiratory distress. Investigation of the relationship between the objective cognitive sequelae of COVID-19 and type of respiratory support received during the acute phase would thus be important to guide both cognitive assessments in long-COVID clinics and the best management strategies for acute COVID-19. Therefore, we explored how two groups of patients who received different respiratory support during COVID-19 differed in their neuropsychological profile as well as the pneumological and motor functions at rehabilitation admission and at discharge, one month later. 

## 2. Materials and Methods

### 2.1. Participants

This observational study was conducted between January and May 2021 including patients with a clinical indication for pulmonary rehabilitation immediately after the acute phase of COVID-19 infection. The study was approved by the local Clinical Scientific Institutes Maugeri’s ethical committee (with Approval Number: 2470, 8 September 2020) and was conducted in accordance with the principles of the Declaration of Helsinki. We obtained informed consent from all participants involved in this study.

**Figure 1:**
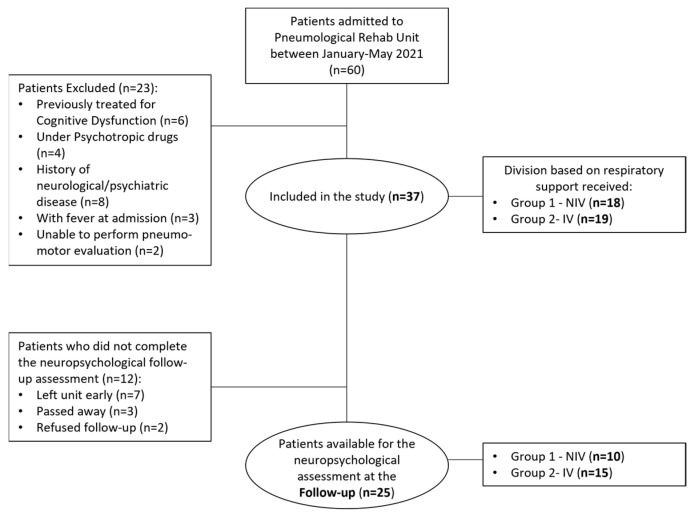
Flow-diagram for the recruitment of patients in the post-COVID cognition assessments.

About 60 patients were admitted to the COVID-19 Pneumological Rehabilitation Unit of the Clinical and Scientific Institutes Maugeri IRCCS Bari, Southern Italy during the above-mentioned period (about 4 months). These patients represented about 80% of the total number of patients who had been hospitalized with COVID-19 in the acute care units of the three main hospitals in Bari and in the province. Out of these 60 patients, 37 (31 male, six females; median age of 67 years, range of 29–88 years) met the inclusion criteria and were included in the study. Inclusion criteria to admit patients in this study were: (1) Previous positive swab and hospitalization for SARS-CoV-2; (2) negative swab at admission to the rehabilitation unit. We excluded from the study patients with fever (*n* = 3), patients who were previously treated for cognitive dysfunctions (*n* = 6), patients receiving psychotropic drugs prior to the hospitalization (*n* = 4), and patients presenting with a history of neurological diseases and/or psychiatric conditions (i.e., dementia of various etiologies, traumatic brain injury, epilepsy, multiple sclerosis, anoxia, a disabling mood disorder, or somatoform disorder, *n* = 8). We also excluded patients unable to perform the pneumological, motor, and neurological exams (*n* = 2). Figure 1 shows the flow-diagram of the sampling and enrollment process.

At admission to the rehabilitation unit, the eligible patients’ information for the following areas was collected. Sociodemographic data such as age, sex, educational level, and clinical information were recorded in the patient file by registered nurses. The medical history of all patients identified previous pathologies. Information regarding smoking status and history of diabetes, obesity, cardiovascular disorders, hypertension, heart disorders or infarcts, chronic obstructive pulmonary disease (COPD), chronic kidney disorder, cancer, stroke, and/or sleep apnea were collected through clinical interviews with all patients. Prior to analysis, data were crosschecked with medical charts and verified by data managers and clinicians for accuracy. These measures were collected within the first week of hospitalization in the rehabilitation unit. 

We divided the sample into two groups based on the type of respiratory support received. Group-1 consisted of patients who received oxygen therapy with Venturi masks or reservoir masks (i.e., low-flow masks that deliver oxygen) or non-invasive mechanical ventilation (i.e., continuous positive airway pressure [CPAP] or bi-level positive airway pressure [BiPAP]). Group-2 consisted of patients who underwent invasive mechanical ventilation (via an endotracheal tube or a tracheostomy tube). Out of the 37 patients, 18 belonged to Group-1 (three females, 15 males; median age 67.5, range 55–88) and 19 belonged to Group-2 (three females, 16 males; median age 63, range 29–78). Participant descriptors, subdivided into groups, are presented in Table 1.

On average, patients were hospitalized for 36.87 days (range: 10–83 days) prior to admission to our rehabilitation unit, with 28 patients arriving from the ICU (mean days in ICU: 28.71). All patients received oxygen therapy for 45.62 days on average. On arrival at our rehabilitation unit, 14 patients (six from ICU) were still receiving oxygen at the time they were tested. The period they spent in our rehabilitation unit was 35 days, corresponding to the length of the rehabilitation program. With respect to neuropsychological assessments, patients underwent initial testing about 2–3 days after admission to the rehabilitation unit and a subset of participants again underwent testing about 1–2 days prior to discharge (approximately one month later). The time between the first symptoms to the first neuropsychological evaluation was on average 142.22 ± 34.5 days, while between the first symptoms and the second neuropsychological evaluation, it was on average 174.81 ± 3.5 days. Concerning the complications during the hospital stay, we found that all patients had pulmonary dysfunctions (i.e., respiratory tract infection, pneumonia, interstitial lung disease, and pulmonary embolism were the most frequent causes of hospitalization); 14/37 patients presented with neuromotor disorders, 2/37 patients had cardiovascular events, and 2/37 patients had renal dysfunction. Twelve out of 37 patients left the rehabilitation unit early against medical advice (*n* = 7), passed away (*n* = 3), or refused to undergo an assessment at discharge (*n* = 2) and were therefore excluded from this follow-up sample. The final follow-up sample consisted of 25 patients (Group-1: *n* = 10: three females, seven males; median age 68.7, range 55–75; Group-2: *n* = 15: two females, 13 males; median age 59.42, range 29–74) who completed rehabilitation and agreed to undergo an assessment at discharge, one month later.

### 2.2. Neuropsychological Data

Patients were assessed by a neuropsychologist who had experience of the cognitive testing of patients with acquired brain injuries. Global cognitive and executive functioning were assessed via administration of the Mini-Mental State Evaluation (MMSE; [31]) and the Frontal Assessment Battery (FAB; [32]), respectively. The purpose of the MMSE is to screen for cognitive impairment (particularly in the elderly), to track cognitive changes that occur with time, and to assess the effects of potential therapeutic drugs on cognitive functioning. It is brief, easily administered, and easily scored. The measure assesses orientation, attention and calculation (serial 7 s, spell “world” backward), language (naming, repetition, comprehension, reading, writing, copying), and immediate and delayed recall. Scores ≥24 indicate normal cognitive status, while lower scores indicate cognitive impairment. FAB is a brief screening battery initially designed as a bedside tool that assesses the presence and severity of executive dysfunction. It involves both cognitive and motor assessments, consisting of tests designed to measure motor programming, conceptualization (abstraction), mental flexibility, sensitivity to interference, inhibitory control, and environmental autonomy. Scores ≥13 indicate normal executive functioning, while lower scores indicate executive impairment. 

The Repeatable Battery for the Assessment of Neuropsychological Status (R-BANS) [33] is a neuropsychological battery composed of two parallel forms (“A” and “B”) of similar difficulty, each divided into 12 subtests, to evaluate five different cognitive domains: immediate and delayed memory, attention, language, visuospatial, and visuo-constructive skills. To make comparisons of test results across age groups, raw scores from the neuropsychological tests were transformed into index scores according to the test manual [34]. Each index score ranges from 40 to 160, with lower scores indicating poorer performance. The total score is the sum of index scores of the five cognitive domains, which are normalized on an age-adjusted scale with a mean of 100 and SD of 15. A R-BANS total score ≤69 is considered indicative of severe cognitive impairment, while scores from 70 to 79 are considered moderate cognitive impairment, and scores from 80 to 89 are considered mild cognitive impairment [34]. R-BANS allows for neuropsychological evaluation in patients aged 20 to 80, affected by head trauma or stroke, or suffering from dementia or psychiatric problems. The R-BANS total score is derived from the scores of the five major indices: immediate memory index, delayed memory index, attention index, language index, and visuospatial/constructional index. The immediate memory index consists of the list learning and story memory subtests; the attention index consists of the digit span and coding subtests; the visuospatial/constructional index consists of the figure copy and line orientation subtests; the language index consists of the picture naming and semantic fluency subtests; and the delayed memory index consists of the list recall, story recall, list recognition, and figure recall subtests. 

R-BANS was used in this study due to two advantages: first, the measure is brief enough that patients can be tested in a relatively short time (30 min), and second, it has two equivalent alternate Italian forms, allowing patients to be tested with equivalent forms at admission to rehabilitation and before discharge. To assess the participants’ affective condition, we administered the Hospital Anxiety and Depression Scale (HADS; [35]). HADS is a simple self-administered questionnaire to establish the presence and severity of anxiety and depression simultaneously, giving separate scores for anxiety and depression. The scale consists of 14 items. Raw scores of 8 to 10 are considered mild, 11 to 15 moderate, and >16 severe. The same cut-off scores were used for the depression and anxiety scales. 

### 2.3. Pneumological and Motor Evaluation

The pulmonary evaluation was performed using a blood gas analysis and pulmonary function tests. Pulmonary function tests were performed using the plethysmograph JAEGER MasterScope Body and interpreted according to the American Thoracic Society/European Respiratory Society (ATS/ERS) guidelines [36,37] in the Lung Function laboratory with the execution of the following maneuvers: forced vital capacity, slow vital capacity, a single breath determination of carbon monoxide uptake. The following spirometric parameters were measured: forced expiratory volume in the first second (FEV1), forced vital capacity (FVC), FEV1/FVC ratio, and diffusing capacity of the lung for carbon monoxide (DLCO) by the single-breath DLCO test. FEV1 is the volume of air that can forcibly be blown out in the first second, after a full inspiration. It is the best reproducible and repeatable parameter of spirometry. FVC is the volume of air that can forcibly be blown out after full inspiration, measured in liters. FVC is also the most basic maneuver in spirometry tests (a test that provides measures of breath). DLCO is the carbon monoxide uptake from a single inspiration in a standard time (usually 10 s). The hemoglobin value was taken to correct the DLCO. All of the variables are usually measured by lung function tests that are interpreted in light of reference values; therefore, each parameter can assume different values based on age, sex, race, height, and weight. All parameters were expressed as absolute values and percentages of the predicted value (%). The Third National Health and Nutrition Examination Survey (NHANES III) reference standard was used [38]. The diffusion deficit was considered as DLCO was less than 80% of the predicted values. Arterial blood gases were assessed on samples from the radial or brachial artery with the patients breathing air in the sitting or semi-orthopneic position for at least 1 h. Arterial oxygen tension (PaO_2_; a measure of the pressure of oxygen dissolved in the blood, which shows how well oxygen moves from the lungs to the bloodstream), the arterial partial pressure of carbon dioxide (PaCO_2_; the amount of carbon dioxide in the blood and how well carbon dioxide can move out of the body), the pH (a measure of the balance of acids and bases in blood, known as blood pH level), and bicarbonate (sHCO_3_^−^ calculated using the measured values of pH and PaCO_2_ to determine the amount of the basic compound made from carbon dioxide) measurements were acquired. 

Motor performance was assessed by the Barthel Index (BI), the 6-m walking test (6MWT), and by neurophysiological examination. BI is a measure of activities of daily living [39]. The total BI score ranges from 0 (maximum level of dependency) to 100 (complete autonomy). A score ≤70 corresponds to severe dependency. The 6MWT [40] was used to evaluate exercise tolerance and was performed according to the ATS practical guidelines [41]. The baseline value of patients unable to perform the test was considered 0 for the analysis. Each patient walked on flat ground as fast as possible without oxygen inhalation and completed the 6MWT independently. The results were expressed as meters and percentages of predicted values calculated using a method described by [42]. From the 6MWT, we obtained the theoretical distance corresponding to the distance that a patient should walk in relation to age, sex, and weight and the distance meters corresponding to the total distance that patients walked during the 6MWT. Finally, from the 6MWT, we also acquired the resting heart rate and the maximum heart rate was monitored using a pulse oximeter (VintusWalk, Vyaire Medical, Inc., New York, NY, USA). Neuromuscular dysfunction was assessed by neurophysiological examination with electromyography and electroneurography. Electromyography with a concentric needle electrode was performed in lower limb muscles in order to detect abnormal spontaneous activity at rest and, if the patients could collaborate, to analyze the presence of myopathic (polyphasic, short duration, low amplitude) motor unit potentials [43]. Motor nerve conduction evaluation was performed in the fibular and tibial nerves of both legs. Sensory nerve conduction evaluation was performed for the superficial peroneal nerve and for the sural nerve in both legs. 

The pulmonary and motor measures listed above were collected by GC, MA, and MG (two clinicians with over ten years of experience in respiratory failure and pulmonary function tests and a certified respiratory physiotherapist) as a part of their typical clinical visit.

### 2.4. Statistical Analysis

The whole sample was divided into two groups according to the respiratory support they received in the acute phase of the disease derived from the clinical data at admission. As aforementioned, Group-1 included patients who received oxygen therapy (with Venturi masks or reservoir masks) or non-invasive mechanical ventilation (CPAP or BiPAP]). Group-2 included patients who underwent invasive mechanical ventilation (via an endotracheal tube or a tracheostomy tube). 

The normality of the distribution for each variable by group was tested using the Shapiro–Wilk test, highlighting a non-normal distribution among most variables. Therefore, a non-parametric approach was chosen to assess the statistical differences by groups. A Wilcoxon’s effect size was used to evaluate the magnitude of any meaningful differences between groups for continuous variables and the prevalence difference was adopted for differences in proportions. We reported *p*-values with a level of significance established at α ≤ 0.05. Statistical significance was inferred from the direction of the upper and lower values of the confidence (whether or not the CI includes zero). Because of the multiple comparisons in this study and the small sample size, a false discovery rate (FDR; [44]) correction was used. The FDR correction is a method to control the number of falsely rejected null hypothesis (Type I error) by establishing the acceptable number of falsely rejected hypotheses from the total number of hypotheses tested (e.g., proportion of false discoveries). The FDR method has shown particular benefit in studies in which multiple comparisons are made with limited sample sizes [34,44,45], as it has consistently been shown to lower the risk of Type I error beyond traditional (e.g., Bonferroni) familywise error correction methods while preventing unduly stringent corrections that might mask truly significant effects (Type II error) [44,45]. While the impact of loss of power is reduced in FDR compared to traditional methods to control for the family wise error rate, there is an interactive effect between the FDR and false non-discovery rate, with increasing emphasis to identify false non-discovery rates (e.g., [34,46]). Full adjusted rank-based regression models were performed on each continuous variable as the dependent variable and the type of respiratory support as the independent variable, adjusted by age, gender, and education level. Spearman non-parametric correlation tests were used to investigate the correlations between variables (Figure S1, Supplementary Materials). Data collected at discharge were compared to data at rehabilitation admission using Wilcoxon’s effect size. All statistical analyses were performed with Rstudio software version 2021.09.1.

## 3. Results

### 3.1. Descriptive Statistics for Patient Groups

Wilcoxon’s effect size analysis did not indicate a significant effect of the main factor education (0.18 (−0.09 to 0.42)) nor for age (0.25 (−0.03 to 0.52)) and sex (0.88 (−22.90 to 24.65)) (see Table 1).

### 3.2. Neuropsychological Measures

**Figure 2:**
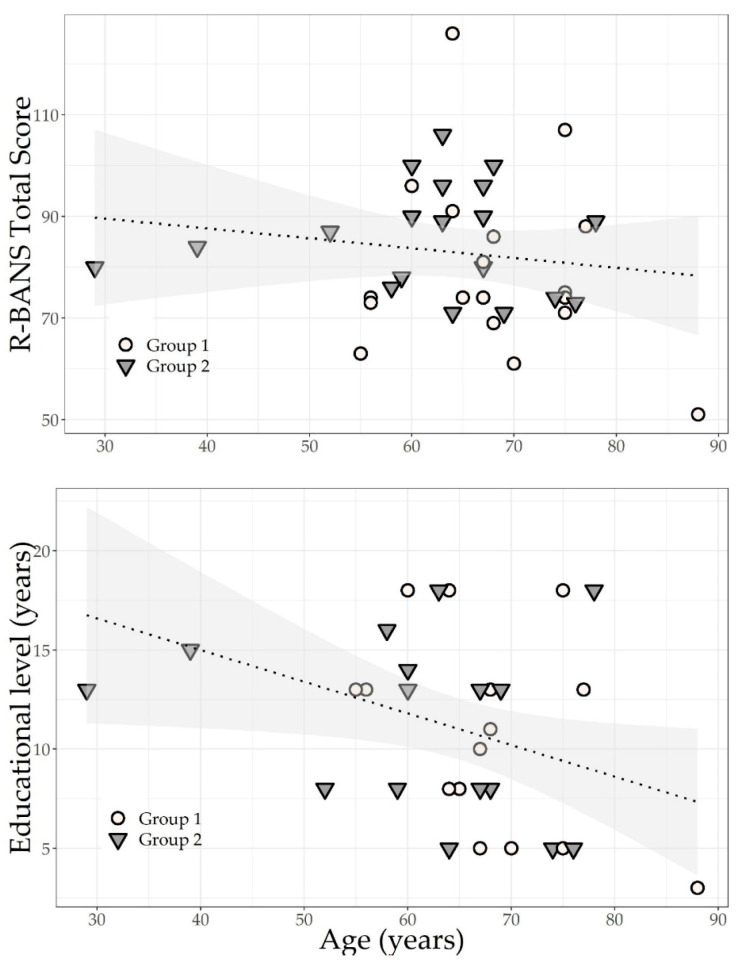
Scatter plots of the Repeatable Battery for the Assessment of Neuropsychological Status (R-BANS) total score according to the two groups studied, their age, and education (Group-1 = without invasive ventilation; Group-2 = with invasive ventilation).

On the MMSE, 8/18 (44%) of patients from Group-1 and 1/19 (5%) of patients from Group-2 presented with global cognitive impairment (mild to severe deficits), as defined by a total score of 23 or below. FAB scores showed that 16/18 (88%) of patients from Group-1 and 5/19 (26%) of patients from Group-2 presented with global executive impairment (mild to severe deficits) as defined by a cut-off score of 12 or below. On the R-BANS, 10 out of 37 patients (27%) demonstrated mild cognitive deficits, 13 out of 37 (35%) showed moderately impaired cognition, and five out of 37 patients (13.5%) showed severe cognitive impairments. Specifically, as seen in Table 2, comparison analyses showed a higher performance of Group-2 compared to Group-1 in R-BANS immediate memory (ES: 0.32, 95% C.I.: 0.03 to 0.62; *p* = 0.04), delayed memory (ES: 0.18, 95% C.I.: 0.07 to 0.63; *p* = 0.03), story recall (ES: 0.31, 95% C.I.: 0.02 to 0.60; *p* = 0.04), figure recall (ES: 0.38, 95% C.I.: 0.10 to 0.69; *p* = 0.05), and coding (ES: 0.32, 95% C.I.: 0.3 to 0.62; *p* = 0.05). In both groups, the R-BANS total scores were significantly correlated with the patients’ age and education (R-BANS: Rho = 0.61 *p* < 0.01, see Figure 2). As measured by the HADS, 10 out of the 18 patients in Group-1 (55%) had mild anxiety and nine out of the 19 patients in Group-2 (47.3%) had mild to moderate anxiety. Eight out of the 18 patients in Group-1 (44%) had mild depression and seven out of the 19 (36.8%) patients in Group-2 had mild depression. 

### 3.3. Pneumological and Motor Measures

With respect to the pneumological data, we found that pO_2_ was significantly higher in Group-1 than Group-2 (ES: 0.43, 95% C.I.: 0.05 to 0.80; *p* < 0.01), as assessed using the Wilcoxon’s effect size. Otherwise, median values of FEV1%, FVC%, DLCO%, paCO_2_, pH, and HCO_3_^−^ were non-significantly lower in Group-2 than in Group-1 (see Table 1). Similarly, the restrictive pattern on spirometry presented with a higher prevalence in Group-2 (75%) compared to Group-1 (53%), but this difference was not meaningful (ES: 21.67, 95% C.I.: −13.5 to 56.8). 

Concerning the motor evaluation, we included a measure of theoretical distance (i.e., the theoretical distance that a patient should walk in relation to age, sex, weight, etc.), distance meters (the total distance that patients walked during a 6-min walk test), score at the Barthel scale (a measure of activities daily living) that includes a Barthel index-dyspnea (indicating how dyspnea precludes or reduces the same activities of daily living), resting heart rate, and maximum heart rate. Neuromuscular dysfunction was assessed to evaluate the presence of the dysfunction of peripheral nerves. 

Concerning the motor parameters, no variables showed a meaningful difference between the two groups, but we found that the percentage of distance meters (median Group-1: 39.5, median Group-2: 33.5; ES: 0.11, 95% C.I.: −0.20 to 0.30) and the score at the Barthel dyspnea scale (median Group-1: 50.5, median Group-2: 41; ES: 0.34, 95% C.I.: −0.02 to 0.70) presented with non-significantly lower median values in Group-2 compared to Group-1. The resting and maximum heart rate presented with non-significantly higher median values in Group-2 (median Group-1: 90, median Group-2: 101; ES: 0.21, 95% C.I.: −0.13 to 0.51 and median Group-1: 106, median Group-2: 112; ES: 0.23, 95% C.I.: −0.03 to 0.51, respectively). Neuromuscular dysfunction was presented and confirmed by electromyography in 10 patients, among which seven (70%) belonged to Group-2, although this was not a statistically significant group difference (ES: 26.57, 95% C.I.: −11.20 to 64.35). 

### 3.4. Examination at Discharge, One Month Later

Subsequently, 25 patients (10 in Group-1, 15 of Group-2) were evaluated at discharge from rehabilitation, approximately one month after admission to the rehabilitation unit. On the R-BANS, five out of 25 patients (2%) demonstrated mild cognitive deficits, seven out of 25 (28%) showed moderately impaired cognition, and one out of 25 patients (4%) showed severe cognitive impairments. Using the R-BANS total scores and appropriate cut-offs, we found that five out of 10 (50%) patients in Group-1 and two out of 15 (13.3%) patients in Group-2 demonstrated mild cognitive deficits, none of the patients in Group-1 (0%), and five out of 15 (33.3%) patients in Group-2 showed moderately impaired cognition, and one out of the 10 patients from Group-1 (10%) and none of the 15 patients from Group-2 (0%) showed severe cognitive impairments. Both groups showed an improvement in scores in several cognitive subdomains between admission and discharge, as displayed in Table 3. Specifically, the improvement was meaningful for R-BANS immediate memory (ES: 0.80, 95% C.I.: 0.62 to 1.03; *p* = 0.04), list learning (ES: 0.72, 95% C.I.: 0.43 to 1.06; *p* = 0.04), delayed memory (ES: 0.62, 95% C.I.: 0.22 to 1.02; *p* = 0.02), figure recall (ES: 0.76, 95% C.I.: 0.52 to 1.04; *p* = 0.02), and coding (ES: 0.57, 95% C.I.: 0.18 to 1.01; *p* = 0.02) for Group-1. Similarly, Group-2 showed a meaningful improvement for R-BANS total score (ES: 0.59, 95% C.I.: 0.26 to 0.94; *p* = 0.04), immediate memory (ES: 0.44, 95% C.I.: 0.05 to 0.83; *p* = 0.04), delayed memory (ES: 0.58, 95% C.I.: 0.22 to 0.95; *p* = 0.02), list recall (ES: 0.48, 95% C.I.: 0.12 to 0.86; *p* = 0.02), and list recognition (ES: 0.62, 95% C.I.: 0.31 to 0.95; *p* = 0.02), attention index score (ES: 0.65, 95% C.I.: 0.36 to 0.94; *p* = 0.02), and coding (ES: 0.43, 95% C.I.: 0.03 to 0.84; *p* = 0.02) at discharge. 

In terms of HADS anxiety scores at discharge (Table 3), three out of the 10 patients of Group-1 (33%) had mild anxiety and four out of the 15 patients of Group-2 (36.3%) had mild to moderate anxiety. Anxiety did not differ between discharge and time of admission in Group-1 (ES: 0.02, 95% C.I.: −0.58 to 0.13; *p* = 0.35) or in Group-2 (ES: 0.67, 95% C.I.: 0.43 to 0.96; *p* = 0.99). Concerning HADS depression scores at discharge, three out of 10 patients (33%) presented mild to moderate depression in Group-1, and only one out of 15 patients in Group-2 presented with mild depression (6%). Depression scores had a meaningful improvement in Group-1 (ES: 0.58, 95% C.I.: 0.13 to 1.04; *p* = 0.04), but not in Group-2 (ES: 0.58, 95% C.I.: 0.25 to 0.92; *p* = 0.98) at discharge assessment. 

With respect to pneumological and motor data, both groups showed an improvement between rehabilitation admission and discharge in several measures, as shown in Table 3. Specifically, Group-1 showed meaningful improvement for FEV1 (ES: 0.51, 95% C.I.: 0.05 to 0.98; *p* = 0.03), FVC (ES: 0.51, 95% C.I.: 0.05 to 0.99; *p* = 0.05), pH (ES: 0.65, 95% C.I.: 0.28 to 1.03; *p* = 0.04), and Barthel Scale (ES: 0.82, 95% C.I.: 0.65 to 1.01; *p* = 0.02). Group 2 showed meaningful improvement for FEV1 (ES: 0.85, 95% C.I.: 0.79 to 0.94; *p* = 0.03), FVC (ES: 0.60, 95% C.I.: 0.29 to 0.96; *p* = 0.04), DLCO (ES: 0.59, 95% C.I.: 0.24 to 0.97; *p* = 0.02), HCO_3−_ (ES: 0.57, 95% C.I.: 0.25 to 0.93; *p* = 0.04), distance meters (ES: 0.70, 95% C.I.: 0.25 to 0.92; *p* = 0.04), and Barthel Scale (ES: 0.88, 95% C.I.: 0.65 to 1.01; *p* = 0.02). 

## 4. Discussion

In this study, we analyzed the neuropsychological, pneumological, and motor data of 37 patients who suffered from COVID-19 who were admitted to our Pneumological Rehabilitation Unit of IRCCS Maugeri Bari, Southern Italy, in the first 4 months of 2021, shortly after the acute phase of their disease. Thus far, to the best of our knowledge, only one study has reported data regarding neuropsychological deficits after COVID-19 in the post-acute phase of the infection in Southern Italy [47], with the majority of other studies being conducted in the north of Italy (i.e., [48,49]). In the present study, we investigated the cognitive profiles of two groups of patients split according to the respiratory support they received in the acute phase of the disease. Group-1 included patients who benefited from oxygen therapy without invasive ventilation (i.e., Venturi masks or reservoir masks, CPAP, or BiPAP). Group-2 included patients who underwent invasive mechanical ventilation (via an endotracheal tube or a tracheostomy tube). 

Most of the respiratory data did not show a meaningful difference between the groups. We only found that patients belonging to Group-1 had higher values of pO_2_ compared to Group-2; this is a value that reflects the amount of oxygen gas dissolved in the blood, indicating the effectiveness of the lungs in pulling oxygen into the blood, allowing for an assumption of less compromised lung function in Group-1. However, we found that Group-1 patients demonstrated greater impairment on neuropsychological assessments compared to Group-2. Interestingly, other studies in the literature have reported similar findings of poorer cognitive functioning in patients who received less invasive oxygen therapy [25,26]. In particular, [26] demonstrated that patients who received invasive respiratory assistance had better cognitive functioning in the sub-acute phase of the disease. The authors suggested that those who underwent non-invasive ventilation treatment would have experienced higher levels of acute and chronic stress compared to those who were intubated and sedated. Presently, the relationship between ventilation therapies in patients who suffered from COVID-19 and their impact on cognition is not yet clear; thus, further investigation is needed. Concerning motor parameters, we did not find any meaningful differences between groups. There was only a trend showing that patients in Group-2 had worse dyspnea values, as measured with the Barthel scale, and lower scores on distance meters (measured by the 6 min walk test), possibly indicative of greater exercise limitation. Overall, these data are in line with the literature [50] and suggest that patients received appropriate respiratory support in the acute phase of the disease.

Concerning cognitive performance, we found that 44% of patients from Group-1 and 5% of patients from Group-2 presented with global cognitive impairment (mild to severe deficits) as measured by the MMSE global score, consistent with previous studies [27,49,51]. Moreover, the FAB scores showed that 88% of patients from Group-1 and 26% of patients from Group-2 presented with global executive impairment (mild to severe deficits), indicating a higher degree of dysexecutive function in Group-1 patients (who received non-invasive mechanical ventilation) compared to Group-2 patients (who benefited from oxygen therapy with masks), in line with previous findings that reported executive function deficits in patients who had contracted COVID-19 [12,52,53] as well as in other acute respiratory syndromes [54]. As suggested by [26], patients who received non-invasive ventilation treatment would have experienced higher levels of acute and chronic stress compared to those who were intubated and sedated, thus resituated a higher degree of executive function deficits. With respect to the R-BANS assessment, we found differences between groups in immediate memory, delayed memory, and coding. Group-2, which presented with higher respiratory effort and lung deficiency, showed statistically significantly better neuropsychological performance in global executive functioning, immediate and delayed memory, and coding compared to Group-1. Recently, a study conducted in Southern Italy investigated the neuropsychological sequelae of moderate to severe SARS-CoV-2 infection in patients within 2 months of negative swab tests [47]. The authors found that 17.4% of patients had severe impairment and 60.13% of patients had mild impairment in short-term and long-term verbal and spatial memory, attention, and executive functions. Similarly, our results documented short-term and long-term verbal and spatial memory and attention impairment. Only two other studies have assessed neuropsychological status by means of R-BANS [55,56]. In a study of patients who survived COVID-19, were discharged home, and followed-up at 4 months in an outpatient service, 46% of the patients demonstrated cognitive impairment, of which the immediate and delayed memory domains were the most affected [30]. Despite the different times of assessment, our findings showed similar results. In a study of Swedish patients who reported persisting symptoms after COVID-19 infection, 37% of patients performed 1.5 SD below the norm in the memory domain, indicating neuropsychological deficits [57]. 

Finally, our neuropsychological data (MMSE, FAB, and R-BANS total scores) significantly correlated with age and education. Although not meaningful, patients in Group-2 were slightly younger and slightly more educated compared to Group-1. At discharge, patients in both groups showed significant cognitive improvement, consistent with some previous findings (e.g., [58]) in immediate memory, delayed memory, and attention (coding). As defined by the R-BANS total scores, 46% of patients in Group-2 still had mild and moderate impairments (no patient showed severe impairments in Group-2 at discharge), and 60% of the patients had mild to severe impairments in Group-1 at discharge. Patients of both groups did not receive psychological support or cognitive training, but only physiotherapy and respiratory rehabilitation. This might explain the improvement registered in Group-1 and Group-2 in several pneumological and motor parameters such as FEV1, FVC, DLCO%, HCO_3−_, and Barthel scale. It is unknown to us whether specific therapies would be enough to recover from cognitive symptoms or whether spontaneous recovery plays a role. On the other hand, data coming from the literature show that early respiratory and motor rehabilitation improves pulmonary symptomatology [59] as well as cognition [60]. Moreover, prior literature on cognitive impairment during inpatient rehabilitation showed that multidimensional cognitive impairment in patients hospitalized for acute respiratory distress syndrome is quite common, and it was found to be persistent in 10% at long-term follow-up [57]. Specifically, the authors found that inpatient rehabilitation exerted significantly positive effects on the patients’ cognitive recovery. Similarly, inpatient rehabilitation can have a positive impact on the cognition and functional outcomes in non-respiratory syndromes (e.g., acquired brain injury [61]). Therefore, we cannot exclude that inpatient rehabilitation had a crucial role in cognitive recovery. 

Concerning mood, the two groups presented with similar degrees of anxiety and depression at admission to the rehabilitation unit. However, as the physical condition ameliorated, a meaningful reduction in depressive symptoms was found only in Group-1 at discharge. Interestingly, despite worse lung damage and higher levels of dyspnea, Group-2 showed improvement in HADS anxiety levels at discharge compared to admission to rehabilitation, while Group-1 showed no change in anxiety scores. As reported by Deng et al. [62], anxiety and depression are frequently experienced during hospitalization. It is assumed that due to the patients’ vulnerability and concern about their clinical condition, such psychiatric symptoms can persist after COVID-19 hospitalization [62]. However, to date, there is limited understanding in the peer-reviewed literature of the duration of emotional distress in patients who had COVID-19. Some authors have found persistence of distress and emotional symptoms after the post-acute phase of infection (i.e., [63]) while others, like the present study, found a resolution of symptomatology after one month (i.e., [64]). 

Limitations should also be acknowledged. The first limitation of our study was that non-hospitalized cases were not included. Moreover, the lack of premorbid neuropsychological evaluation constitutes a missing starting point of the patients’ clinical path that would have allowed a better understanding of the impact of COVID-19 on cognition. Nonetheless, patients who stayed in an ICU may develop memory and executive disorders [57]; however, we did not include in our study patients who had stayed in the ICU for reasons other than COVID-19. Thus, future studies should investigate to what degree patients who had stayed in the ICU for non-COVID-19 reasons differed in their neuropsychological profiles from patients who had had COVID-19 and required ICU admission. Furthermore, we acknowledge that our study cannot be used to draw conclusions about the generalizability of post-COVID cognitive impairments in a wider population. This study was based on a small convenience sample of patients who entered our rehabilitation unit during a critical period of the pandemic. Our study should be considered preliminary in nature and could be considered as a pilot study, suggestive of a trend in the population. Hence, we may have overestimated the effects we found, and the results should not be considered in terms of prediction. Multivariable models should be considered as the best statistical approach to measure the effect of association of the variables considered among the two groups, and to assess the prediction power and goodness of fit in the multivariable model. This approach could not be used in this analysis due to the large number of variables included in the analysis with respect to the number of observations/patients included in the small sample. Conversely, a larger study would have required more funding and personnel resources that were not available at that point in the COVID-19 pandemic. Finally, our findings indicate the importance of an extended neuropsychological evaluation to identify and quantify cognitive deficits for better classification of the COVID-19 cognitive profile. 

## 5. Conclusions

This study was conducted in a rehabilitation setting on 37 patients hospitalized after COVID-19-negative swabs. We studied the cognitive, pneumological, and motor sequelae according to the respiratory support that patients received during their COVID-19 infection. Overall, about 75% of our patients presented with mild to severe cognitive deficits, as measured by the R-BANS total score at admission to the rehabilitation unit during the subacute phase of the disease, suggesting the link between cognitive deficits and restricted oxygen delivery to the brain. In particular, our findings showed that patients who benefited from oxygen therapy with masks (Group-1) presented a higher degree of dysexecutive function, immediate and delayed memory, and attention coding task compared to patients who received invasive mechanical ventilation (Group-2). Though interpretation is limited by the lack of a comparator group and by the small sample sizes, this finding can be considered as an early benchmark, suggesting that patients who received oxygen therapy with masks experienced higher levels of acute and chronic stress compared to those who benefitted from invasive mechanical ventilation, notwithstanding the latter treatment being more aggressive. In the future, we need further investigations into the relationship between ventilation therapies and the neuropsychological profiles of COVID-19 with larger samples. Concerning the pneumological and motor parameters, we did not find any significant differences between the groups. Finally, despite patients showing a meaningful improvement at discharge one month later, cognitive impairment persisted in a greater number of patients; therefore, our data also suggest the need for long-term neuropsychological follow-up and treatment targeting executive functions, memory, and attention for patients coming from the post-acute phase of COVID-19.

## Figures and Tables

**Table 1:** Description of the whole sample according to the type of respiratory support (*n* = 37).

	Group-1 (Without Invasive Ventilation)	Group-2 (With Invasive Ventilation)	Effect Size *	*p*^§^ 0.05
	Median (min to max)	Median (min to max)		
	Sociodemographic and Clinical Data			
Proportions (%)	18 (48.60)	19 (51.40)		
Age (years)	67.5 (55 to 88)	63 (29 to 78)	0.25 (−0.11 to 0.61)	0.06
Sex				
Female	3 (16.70)	3 (15.80)	0.88 (−30.37 to 32.12)	0.64
Male	15 (83.30)	16 (84.20)		
Educational level	10.5 (3 to 18)	13 (5 to 18)	0.18 (−0.18 to 0.49)	0.86
BMI (Kg/m^2^)	27.8 (22.3 to 33.8)	28.05 (22.8 to 37.1)	0.01 (−0.43 to 0.15)	0.50
Smoking status				
Ex-smokers	8 (44.40)	8 (42.10)	2.30 (−13.26 to 7.60)	0.57
Current smokers	10 (55.60)	11 (57.90)		
Time of positivity (days)	32 (12 to 62)	40 (5 to 59)	0.24 (−0.13 to 0.61)	0.93
COPD	2 (11.10)	2 (10.50)	−0.58 (−26.91 to 25.74)	0.71
Carcinoma	1 (5.60)	4 (21.10)	15.50 (−12.32 to 43.31)	0.18
Cardiovascular diseases	8 (44.40)	5 (26.30)	−18.13 (−57.97 to 21.71)	0.93
Chronic kidney disease	6 (33.30)	3 (15.80)	−17.54 (−53.37 to 18.38)	0.95
Hypertension	11 (61.10)	10 (52.60)	−8.48 (−50.27 to 33.31)	0.80
Obesity	8 (44.40)	5 (26.30)	−18.13 (−57.97 to 21.71)	0.93
Apneas	1 (5.60)	2 (10.50)	4.97 (−17.88 to 27.82)	0.53
Diabetes	9 (50.00)	5 (26.30)	−23.68 (−63.67 to 16.30)	0.96
	Pneumological and Motor Evaluation			
FEV1 %	84 (41 to 102)	75 (57 to 138)	0.18 (−0.25 to 0.55)	0.17
FVC %	76 (34 to 100)	67 (40 to 129)	0.16 (−0.24 to 0.49)	0.21
FEV1/FVC ratio	1.08 (0.91 to 1.29)	1.07 (0.92 to 1.17)	0.06 (−0.36 to 0.29)	0.37
DLCO %	55 (34 to 81)	48 (1 to 63)	0.24 (−0.19 to 0.64)	0.12
HCO_3_^−^	26 (24 to 49)	25.5 (22 to 30)	0.02 (−0.47 to 0.20)	0.54
paCO_2_	36.5 (25 to 71)	37 (30 to 46)	0.05 (−0.37 to 0.23)	0.60
pH	7.44 (7.39 to 7.49)	7.44 (7.36 to 7.49)	0.20 (−0.20 to 0.56)	0.87
pO_2_	80 (54 to 126)	70 (44 to 81)	**0.43 (0.05 to 0.80)**	**<0.01**
Spirometry pattern	53%	75%	21.67 (−13.5 to 56.8)	0.28
Theoretical distance %	39.5 (12 to 66)	33.5 (21 to 52)	0.11 (−0.14 to 0.67)	0.92
Distance meters	185 (60 to 385)	200 (120 to 240)	0.03 (−0.37 to 0.23)	0.59
Barthel Scale	50.50 (13 to 100)	41 (5 to 60)	0.34 (−0.13 to 0.81)	0.20
Heart rate (rest)	90 (58 to 123)	101 (72 to 120)	0.21 (−0.20 to 0.58)	0.87
Heart rate (maximum)	106.5 (88 to 124)	112 (98 to 139)	0.23 (−0.17 to 0.61)	0.89
Neuromuscular dysfunction	3 (27.30)	7 (53.80)	26.57 (−23.07 to 76.22)	0.18

Notes. Bolded and underlined text corresponds to a meaningful/statistically significant finding. All data are shown as the median (range) for continuous variables and as *n* (%) for proportions. * Wilcoxon’s effect size for the continuous variables and prevalence differences for the proportions. ^§^ Wilcoxon rank sum test for dependent samples (conf. level: 97.5, one-tailed). BMI: body mass index, COPD: chronic obstructive pulmonary disease, DLCO: transfer factor for carbon monoxide, FEV: forced expiratory volume, FVC: forced vital capacity. Time of positivity refers to the time between the first positive swab for COVID-19 and the first negative swab.

**Table 2:** Neuropsychological, pneumological, and motor data for the two samples (*n* = 37).

	Group-1 (Without Invasive Ventilation)	Group-2 (With Invasive Ventilation)				
	Median (Range)	Median (Range)	Effect Size *	p (FDR)	Coefficients (95%) **	Stand. Error
	Neuropsychological data					
MMSE	26.5 (20 to 30)	29 (19 to 30)	0.29 (−0.01 to 0.58)	0.25	−1.01 (−2.46 to 0.43)	0.74
FAB	10.50 (5 to 17)	14 (3 to 16)	**0.39 (0.10 to 0.70)**	**0.02**	−1.67 (−3.07 to −0.27)	0.71
HADS Anxiety	8.5 (0 to 17)	7 (4 to 15)	0.06 (−0.23 to 0.18)	0.72	−0.90 (−3.91 to 2.11)	1.53
HADS Depression	6 (0 to 15)	6 (2 to 16)	0.03 (−0.28 to 0.12)	0.89	−1.13 (−3.93 to 1.70)	1.44
R−BANS Total Score	74 (51 to 126)	87 (71 to 106)	0.28 (−0.02 to 0.57)	0.09	−4.34 (−10.89 to 2.21)	3.34
R−BANS Immediate Memory	81 (62 to 122)	93 (59 to 109)	**0.32 (0.03 to 0.62)**	**0.04**	−8.33 (−16.99 to 0.19)	4.38
Story memory	14.5 (5 to 20)	17 (1 to 24)	0.25 (−0.03 to 0.53)	0.10	−1.27 (−3.97 to 1.42)	1.37
List learning	21 (15 to 35)	25 (17 to 34)	0.30 (−0.01 to 0.60)	0.14	**−2.71 (−5.39 to −0.03)**	**1.36**
R−BANS Delayed Memory	86 (63 to 146)	99 (79 to 128)	**0.18 (0.07 to 0.63)**	**0.03**	**−10.61 (−21.21 to −0.01)**	**5.40**
Story Recall	6.5 (0 to 11)	8 (3 to 12)	**0.31 (0.02 to 0.60)**	**0.04**	−1.01 (−2.71 to 0.68)	0.86
List Recall	4 (0 to 9)	6 (0 to 8)	0.23 (−0.04 to 0.50)	0.14	−1.10 (−3.05 to 0.83)	0.99
List Recognition	17 (10 to 20)	18 (13 to 20)	0.23 (−0.05 to 0.51)	0.06	−0.59 (−1.84 to 0.65)	0.63
Figure Recall	10 (5 to 20)	13 (0 to 20)	**0.38 (0.10 to 0.69)**	**0.05**	−2.04 (−4.52 to 0.43)	1.26
R−BANS Attention	77.5 (45 to 133)	85 (65 to 120)	0.18 (−0.08 to 0.42)	0.25	36.45 (−14.20 to 87.11)	6.35
Coding	20 (6 to 55)	33 (0 to 51)	**0.32 (0.03 to 0.62)**	**0.05**	**−6.80 (−12.25 to −1.34)**	**2.78**
Digit Span	7 (4 to 12)	8 (5 to 13)	0.09 (−0.19 to 0.26)	0.46	−0.34 (−1.67 to 0.98)	0.67
R−BANS Language	79.5 (57 to 103)	77 (69 to 111)	0.02 (−0.29 to 0.09)	0.92	3.25 (−5.69 to 7.05)	3.25
Naming	10 (8 to 10)	10 (9 to 10)	0.04 (−0.25 to 0.15)	0.45	10.00 (−503.23 to 523.23)	261.85
Semantic Fluency	14.5 (6 to 26)	16 (7 to 21)	0.10 (−0.16 to 0.28)	0.53	0.01 (−3.07 to 3.07)	1.56
R−BANS Visuospatial/Construction	88 (58 to 120)	99 (62 to 123)	0.16 (−0.12 to 0.39)	0.33	−4.07 (−16.34 to 8.20)	6.26
Figure Copy	14.5 (10 to 20)	18 (0 to 20)	0.20 (−0.07 to 0.47)	0.22	−0.91 (−3.57 to 1.74)	1.35
Line Orientation	14.5 (3 to 18)	13 (6 to 20)	0.12 (−0.15 to 0.31)	0.29	−0.04 (−3.00 to 2.91)	1.50
	Pneumological and Motor data					
Theoretical distance (%)	39.5 (12 to 66)	33.5 (21 to 52)	0.11 (−0.14 to 0.67)	0.15	0.30 (−17.76 to 18.37)	9.21
Distance Meters	185 (60 to 385)	200 (120 to 240)	0.03 (−0.31 to 0.16)	0.84	−5.23 (−64.00 to 53.53)	29.89
Barthel Scale	50.50 (13 to 100)	41 (5 to 60)	0.34 (−0.02 to 0.70)	0.15	15.22 (−9.13 to 39.59)	15.22
FEV (%)	84 (41 to 102)	75 (57 to 138)	0.18 (−0.13 to 0.46)	0.35	2.91 (−6.19 to 12.02)	4.64
FVC (%)	76 (34 to 100)	67 (40 to 129)	0.16 (−0.16 to 0.40)	0.42	3.35 (−9.89 to 16.61)	0.38
DLCO (%)	55 (34 to 81)	48 (1 to 63)	0.24 (−0.11 to 0.55)	0.24	4.43 (−7.83 to 16.70)	6.26
HCO_3−_	26 (24 to 49)	25.5 (22 to 30)	0.02 (−0.39 to 0.12)	0.95	−0.73 (−3.57 to 2.09)	1.44
paCO_2_	36.5 (25 to 71)	37 (30 to 46)	0.05 (−0.29 to 0.16)	0.81	−0.33 (−3.84 to 3.16)	1.78
pO_2_	80 (54 to 126)	70 (44 to 81)	**0.43 (0.14 to 0.73)**	**0.01**	**13.02 (3.31 to 22.74)**	4.95
pH	7.44 (7.39 to 7.49)	7.44 (7.36 to 7.49)	0.20 (−0.10 to 0.48)	0.27	−0.02 (−0.04 to 0.01)	0.01

Notes. Bolded and underlined text corresponds to a meaningful/statistically significant finding. * Wilcoxon’s effect size for continuous variables and prevalence differences for proportions. ** Rank based estimates of the regression coefficients of each continuous variable as the dependent variable and type of treatment (invasive ventilation as the base) as a regressor adjusted for age, sex, and educational level. MMSE: Mini Mental State Examination, FAB: Frontal Assessment Battery, HADS: Hamilton Anxiety and Depression Scale, R-BANS: Repeatable Battery for the Assessment of Neuropsychological Status.

**Table 3:** Description of neuropsychological, pneumological, and motor assessments before and after treatment. *n* = 25.

	Group 1				Group 2			
	Before	After	Wilcoxon’s Effect Size	*p* (FDR)	Before	After	Wilcoxon’s Effect Size	*p* (FDR)
Proportions (%)	10 (50.00)	10 (50.00)			15 (50.00)	15 (50.00)		
	Neuropsychological Assessment							
HADS/A	6 (2 to 15)	6 (2 to 16)	0.02 (−0.58 to 0.13)	0.35	8 (4 to 15)	6 (2 to 10)	**0.67 (0.43 to 0.96)**	**0.02**
HADS/D	5.5 (2 to 15)	5.5 (1 to 11)	**0.58 (0.13 to 1.04)**	**0.05**	6 (2 to 16)	4 (1 to 10)	**0.58 (0.25 to 0.92)**	**0.05**
R−BANS Total Score	78 (63 to 107)	77 (66 to 108)	0.40 (−0.04 to 0.84)	0.24	84 (71 to 106)	89 (71 to 121)	**0.59 (0.26 to 0.94)**	**0.04**
R−BANS Immediate Memory	79.5 (62 to 109)	88 (78 to 114)	**0.80 (0.62 to 1.03)**	**0.04**	93 (59 to 109)	97 (76 to 115)	**0.44 (0.05 to 0.83)**	**0.04**
Story Memory	14.5 (10 to 20)	16 (8 to 21)	0.14 (−0.39 to 0.38)	0.34	17 (1 to 24)	18 (15 to 23)	0.20 (−0.20 to 0.49)	0.34
List Learning	19.5 (17 to 28)	25.5 (18 to 31)	**0.72 (0.43 to 1.06)**	**0.04**	25 (17 to 34)	28 (15 to 35)	0.42 (−0.04 to 0.86)	0.44
R−BANS Delayed Memory	92.5 (63 to 109)	102 (72 to 119)	**0.62 (0.22 to 1.02)**	**0.02**	95 (79 to 128)	101 (83 to 128)	**0.58 (0.22 to 0.95)**	**0.02**
Story Recall	7.5 (6 to 11)	8 (3 to 12)	0.26 (−0.23 to 0.61)	0.94	9 (3 to 12)	10 (5 to 11)	0.31 (−0.09 to 0.68)	0.46
List Recall	4 (0 to 7)	4.5 (0 to 9)	**0.52 (0.06 to 0.97)**	0.32	6 (0 to 8)	6 (3 to 9)	**0.48 (0.12 to 0.86)**	**0.02**
List Recognition	17.5 (15 to 19)	18 (10 to 20)	0.14 (−0.39 to 0.40)	0.23	18 (13 to 20)	19 (16 to 20)	**0.62 (0.31 to 0.95)**	**0.02**
Figure Recall	11 (6 to 13)	14 (10 to 17)	**0.76 (0.52 to 1.04)**	**0.02**	13 (0 to 20)	13 (8 to 20)	0.28 (−0.11 to 0.62)	0.34
R−BANS Attention	80 (62 to 108)	81 (62 to 115)	0.42 (−0.09 to 0.9)	0.23	85 (70 to 120)	96 (72 to 132)	**0.65 (0.36 to 0.94)**	**0.02**
Coding	20 (16 to 44)	28.5 (13 to 41)	**0.57 (0.18 to 1.01)**	**0.02**	33 (0 to 50)	39 (15 to 54)	**0.43 (0.03 to 0.84)**	**0.02**
Digit Span	7 (6 to 11)	7 (6 to 13)	0.01 (−0.50 to 1.50)	0.34	8 (5 to 13)	10 (6 to 12)	0.25 (−0.18 to 0.60)	0.34
R−BANS Language	79.5 (61 to 89)	80 (76 to 90)	0.11 (−0.43 to 0.33)	0.76	78 (69 to 111)	80 (69 to 108)	0.20 (−0.21 to 0.50)	0.76
Naming	10 (8 to 10)	10 (10 to 10)	0.01 (−0.50 to 1.50)	0.89	10 (9 to 10)	10 (9 to 10)	0.01 (−0.50 to 1.50)	0.89
Semantic Fluency	14 (6 to 21)	13.5 (6 to 22)	0.40 (−0.11 to 0.86)	0.85	16 (10 to 21)	14 (8 to 28)	0.18 (−0.25 to 0.47)	0.85
R−BANS Visuospatial/Construction	94 (58 to 119)	88.5 (75 to 106)	0.11 (−0.41 to 0.32)	0.73	98 (62 to 118)	93 (64 to 112)	0.11 (−0.34 to 0.32)	0.73
Figure Copy	15 (10 to 19)	13.5 (7 to 19)	0.42 (−0.06 to 0.86)	0.98	18 (0 to 20)	16 (2 to 19)	0.19 (−0.24 to 0.48)	0.98
Line Orientation	16.5 (3 to 18)	15 (12 to 18)	0.22 (−0.28 to 0.56)	0.45	15 (6 to 20)	17 (11 to 19)	0.26 (−0.14 to 0.60)	0.45
	Pneumological and Motor Evaluations							
FEV1 %	89.5 (8)	77 (25.25)	**0.51 (0.05 to 0.98)**	**0.03**	90 (11.5)	70 (11.5)	**0.85 (0.79 to 0.94)**	**0.03**
FVC %	83 (10)	65 (23.75)	**0.51 (0.05 to 0.99)**	**0.05**	85 (7.5)	64 (18)	**0.60 (0.29 to 0.96)**	**0.04**
FEV1/FVC ratio	67 (232)	231 (90.75)	0.40 (−0.11 to 0.86)	0.79	33 (284)	222 (53)	**0.60 (0.28 to 0.94)**	**0.08**
DLCO %	48 (23)	46.5 (18.75)	0.14 (−0.41 to 0.39)	0.54	52 (24)	40 (11)	**0.59 (0.24 to 0.97)**	**0.02**
HCO_3−_	25 (1.75)	26 (1.88)	0.06 (−0.51 to 0.23)	0.83	25 (4)	26 (3)	**0.57 (0.25 to 0.93)**	**0.04**
paCO_2_	38 (3.25)	39 (6.5)	0.17 (−0.38 to 0.47)	0.84	38 (4.5)	38 (3.5)	0.31 (−0.09 to 0.70)	0.84
pH	7.44 (0.03)	7.43 (0.04)	**0.65 (0.28 to 1.03)**	**0.04**	7.45 (365.82)	7.48 (730.06)	0.36 (−0.05 to 0.75)	0.56
pO_2_	74 (5.25)	77 (14.75)	0.32 (−0.13 to 0.72)	0.98	76 (13.5)	70 (16.5)	0.38 (−0.01 to 0.79)	0.68
Theoretical distance%	511 (72.5)	545 (57.5)	0.11 (−0.13 to 0.80)	0.89	510 (76.5)	510 (74)	−	0.89
Distance meters	205 (75)	150 (105)	0.46 (−0.04 to 0.93)	0.45	240 (35)	180 (35)	**0.70 (0.25 to 0.92)**	**0.04**
Barthel Scale	27.5 (20.75)	57.5 (18.5)	**0.82 (0.65 to 1.01)**	**0.02**	12 (17.5)	52 (12)	**0.88 (0.65 to 1.01)**	**0.02**
Heart rate (rest)	92.5 (26.25)	90 (14)	0.18 (−0.31 to 0.46)	0.44	95 (3)	85 (13.5)	0.39 (−0.07 to 0.81)	0.44
Heart rate (maximum)	112.5 (14.75)	104 (15.25)	**0.65 (0.23 to 1.06)**	0.65	109 (1.5)	101.5 (11.5)	0.31 (−0.16 to 0.72)	0.65

Notes. Bolded and underlined text corresponds to a meaningful/statistically significant finding. Paired Wilcoxon’s effect size. HADS: Hamilton Anxiety and Depression Scale, HADS-A: Anxiety; HADS-D: Depression; R-BANS: Repeatable Battery for the Assessment of Neuropsychological Status.

## Data Availability

Upon request to the corresponding author.

## Supplementary Materials

The following supporting information can be downloaded at: https://www.mdpi.com/article/10.3390/brainsci13010084/s1, Figure S1: Spearman non-parametric correlation tests used to investigate the correlations between variables.

## References

[1] Solomon T. (2021). Neurological Infection with SARS-CoV-2—The Story so Far. Nat. Rev. Neurol..

[2] Subbarao K., Mahanty S. (2020). Respiratory Virus Infections: Understanding COVID-19. Immunity.

[3] Al-Ramadan A., Rabab’h O., Shah J., Gharaibeh A. (2021). Acute and Post-Acute Neurological Complications of COVID-19. Neurol. Int..

[4] Ferrario S.R., Panzeri A., Cerutti P., Sacco D. (2021). The Psychological Experience and Intervention in Post-Acute COVID-19 Inpatients. Neuropsychiatr. Dis. Treat.

[5] Khatoon F., Prasad K., Kumar V. (2020). Neurological Manifestations of COVID-19: Available Evidences and a New Paradigm. J. Neurovirol..

[6] Miners S., Kehoe P.G., Love S. (2020). Cognitive Impact of COVID-19: Looking beyond the Short Term. Alzheimer’s Res. Ther..

[7] Steardo L., Steardo L., Verkhratsky A. (2020). Psychiatric Face of COVID-19. Transl. Psychiatry.

[8] Almeria M., Cejudo J.C., Sotoca J., Deus J., Krupinski J. (2020). Cognitive Profile Following COVID-19 Infection: Clinical Predictors Leading to Neuropsychological Impairment. Brain Behav. Immun. Health.

[9] Llach C.D., Vieta E. (2021). Mind Long COVID: Psychiatric Sequelae of SARS-CoV-2 Infection. Eur. Neuropsychopharmacol..

[10] Rogers J.P., Chesney E., Oliver D., Pollak T.A., McGuire P., Fusar-Poli P., Zandi M.S., Lewis G., David A.S. (2020). Psychiatric and Neuropsychiatric Presentations Associated with Severe Coronavirus Infections: A Systematic Review and Meta-Analysis with Comparison to the COVID-19 Pandemic. Lancet Psychiatry.

[11] Monti G., Leggieri C., Fominskiy E., Scandroglio A.M., Colombo S., Tozzi M., Moizo E., Mucci M., Crivellari M., Pieri M. (2021). Two-Months Quality of Life of COVID-19 Invasively Ventilated Survivors; an Italian Single-Center Study. Acta Anaesthesiol. Scand..

[12] Jaywant A., Vanderlind W.M., Alexopoulos G.S., Fridman C.B., Perlis R.H., Gunning F.M. (2021). Frequency and Profile of Objective Cognitive Deficits in Hospitalized Patients Recovering from COVID-19. Neuropsychopharmacology.

[13] Ferrucci R., Dini M., Groppo E., Rosci C., Reitano M.R., Bai F., Poletti B., Brugnera A., Silani V., Monforte A.D. (2021). Long-Lasting Cognitive Abnormalities after COVID-19. Brain Sci..

[14] Miskowiak K.W., Johnsen S., Sattler S.M., Nielsen S., Kunalan K., Rungby J., Lapperre T., Porsberg C.M. (2021). Cognitive Impairments Four Months after COVID-19 Hospital Discharge: Pattern, Severity and Association with Illness Variables. Eur. Neuropsychopharmacol..

[15] Tavares-Júnior J.W.L., de Souza A.C.C., Borges J.W.P., Oliveira D.N., Siqueira-Neto J.I., Sobreira-Neto M.A., Braga-Neto P. (2022). COVID-19 Associated Cognitive Impairment: A Systematic Review. Cortex.

[16] Hadad R., Khoury J., Stanger C., Fisher T., Schneer S., Ben-Hayun R., Possin K., Valcour V., Aharon-Peretz J., Adir Y. (2022). Cognitive Dysfunction Following COVID-19 Infection. J. Neurovirol..

[17] Sartori A.C., Vance D.E., Slater L.Z., Crowe M. (2012). The Impact of Inflammation on Cognitive Function in Older Adults: Implications for Healthcare Practice and Research. J. Neurosci. Nurs..

[18] Warren-Gash C., Forbes H.J., Williamson E., Breuer J., Hayward A.C., Mavrodaris A., Ridha B.H., Rossor M.N., Thomas S.L., Smeeth L. (2019). Human Herpesvirus Infections and Dementia or Mild Cognitive Impairment: A Systematic Review and Meta-Analysis. Sci. Rep..

[19] Antonelli-Incalzi R., Corsonello A., Trojano L., Acanfora D., Spada A., Izzo O., Rengo F. (2008). Correlation between Cognitive Impairment and Dependence in Hypoxemic COPD. J. Clin. Exp. Neuropsychol..

[20] Herridge M.S., Moss M., Hough C.L., Hopkins R.O., Rice T.W., Bienvenu O.J., Azoulay E. (2016). Recovery and Outcomes after the Acute Respiratory Distress Syndrome (ARDS) in Patients and Their Family Caregivers. Intensive Care Med..

[21] Wang J., Song R., Dove A., Qi X., Ma J., Laukka E.J., Bennett D.A., Xu W. (2022). Pulmonary Function Is Associated with Cognitive Decline and Structural Brain Differences. Alzheimer’s Dement..

[22] Vitacca M., Carone M., Clini E.M., Paneroni M., Lazzeri M., Lanza A., Privitera E., Pasqua F., Gigliotti F., Castellana G. (2020). Joint Statement on the Role of Respiratory Rehabilitation in the COVID-19 Crisis: The Italian Position Paper. Respiration.

[23] Arenivas A., Carter K.R., Harik L.M., Hays K.M. (2020). COVID-19 Neuropsychological Factors and Considerations within the Acute Physical Medicine and Rehabilitation Setting. Brain Inj..

[24] Simpson R., Robinson L. (2020). Rehabilitation after Critical Illness in People with COVID-19 Infection. Am. J. Phys. Med. Rehabil..

[25] Alemanno F., Houdayer E., Parma A., Spina A., del Forno A., Scatolini A., Angelone S., Brugliera L., Tettamanti A., Beretta L. (2021). COVID-19 Cognitive Deficits after Respiratory Assistance in the Subacute Phase: A COVID Rehabilitation Unit Experience. PLoS One.

[26] Manera M.R., Fiabane E., Pain D., Aiello E.N., Radici A., Ottonello M., Padovani M., Wilson B.A., Fish J., Pistarini C. (2022). Clinical Features and Cognitive Sequelae in COVID-19: A Retrospective Study on N=152 Patients. Neurol. Sci..

[27] Woo M.S., Malsy J., Pöttgen J., Seddiq Zai S., Ufer F., Hadjilaou A., Schmiedel S., Addo M.M., Gerloff C., Heesen C. (2020). Frequent Neurocognitive Deficits after Recovery from Mild COVID-19. Brain Commun..

[28] Fotuhi M., Mian A., Meysami S., Raji C.A. (2020). Neurobiology of COVID-19. J. Alzheimer’s Dis..

[29] Holmes E.A., O’Connor R.C., Perry V.H., Tracey I., Wessely S., Arseneault L., Ballard C., Christensen H., Cohen Silver R., Everall I. (2020). Multidisciplinary Research Priorities for the COVID-19 Pandemic: A Call for Action for Mental Health Science. Lancet Psychiatry.

[30] Alnefeesi Y., Siegel A., Lui L.M.W., Teopiz K.M., Ho R.C.M., Lee Y., Nasri F., Gill H., Lin K., Cao B. (2021). Impact of SARS-CoV-2 Infection on Cognitive Function: A Systematic Review. Front. Psychiatry.

[31] Measso G., Cavarzeran F., Zappala G., Lebowitz B.D., Crook T.H., Pirozzolo F.J., Amaducci L.A., Massari Fidia SpA D., Terme A., Francesco Grigoletto I. (1993). The Mini-Mental State Examination: Normative Study of an Italian Random Sample. Dev. Neuropsychol..

[32] Appollonio I., Leone M., Isella V., Piamarta F., Consoli T., Villa M.L., Forapani E., Russo A., Nichelli P. (2005). The Frontal Assessment Battery (FAB): Normative Values in an Italian Population Sample. Neurol. Sci..

[33] Ponteri M., Pioli R., Padovani A., Tunesi S., de Girolamo G. (2007). The Repeatable Battery for the Assessment of Neuropsychological Status (RBANS): Adattamento Italiano.

[34] Schoenberg M.R., Rinehardt E., Duff K., Mattingly M., Bharucha K.J., Scott J.G. (2012). Assessing Reliable Change Using the Repeatable Battery for the Assessment of Neuropsychological Status (RBANS) for Patients with Parkinson’s Disease Undergoing Deep Brain Stimulation (DBS) Surgery. Clin. Neuropsychol..

[35] Iani L., Lauriola M., Costantini M. (2014). A Confirmatory Bifactor Analysis of the Hospital Anxiety and Depression Scale in an Italian Community Sample. Health Qual. Life Outcomes.

[36] Graham B.L., Brusasco V., Burgos F., Cooper B.G., Jensen R., Kendrick A., Macintyre N.R., Thompson B.R., Wanger J. (2017). 2017 ERS/ATS Standards for Single-Breath Carbon Monoxide Uptake in the Lung. Eur. Respir. J..

[37] Graham B.L., Steenbruggen I., Barjaktarevic I.Z., Cooper B.G., Hall G.L., Hallstrand T.S., Kaminsky D.A., McCarthy K., McCormack M.C., Miller M.R. (2019). Standardization of Spirometry 2019 Update an Official American Thoracic Society and European Respiratory Society Technical Statement. Am. J. Respir. Crit. Care Med..

[38] Pellegrino R., Viegi G., Brusasco V., Crapo R.O., Burgos F., Casaburi R., Coates A., van der Grinten C.P.M., Gustafsson P., Hankinson J. (2005). Interpretative Strategies for Lung Function Tests. Eur. Respir. J..

[39] Shah S., Cooper B. (1989). Improving the Sensitivity of the Barthel Index for Stroke Rehabilitation. J. Clin. Epidemiol..

[40] Holland A.E., Spruit M.A., Troosters T., Puhan M.A., Pepin V., Saey D., McCormack M.C., Carlin B.W., Sciurba F.C., Pitta F. (2014). An Official European Respiratory Society/American Thoracic Society Technical Standard: Field Walking Tests in Chronic Respiratory Disease. Eur. Respir. J..

[41] ATS Committee on Proficiency Standards for Clinical Pulmonary Function Laboratories (2002). American Thoracic Society ATS Statement: Guidelines for the Six-Minute Walk Test. Am. J. Respir. Crit. Care Med..

[42] Enright P.L., Sherrill D.L. (1998). Reference Equations for the Six-Minute Walk in Healthy Adults. Am. J. Respir. Crit. Care Med..

[43] Lacomis D. (2000). The Use of Percutaneous Needle Muscle Biopsy in the Diagnosis of Myopathy. Curr. Rheumatol. Rep..

[44] Benjamini Y., Hochberg Y. (1995). Controlling the False Discovery Rate: A Practical and Powerful Approach to Multiple Testing. J. R. Stat. Soc. Ser. B.

[45] Glickman M.E., Rao S.R., Schultz M.R. (2014). False Discovery Rate Control Is a Recommended Alternative to Bonferroni-Type Adjustments in Health Studies. J. Clin. Epidemiol..

[46] Genovese C., Wasserman L. (2002). Operating Characteristics and Extensions of the False Discovery Rate Procedure. J. R. Stat. Soc. Ser. B.

[47] Moretta P., Ambrosino P., Lanzillo A., Marcuccio L., Fuschillo S., Papa A., Santangelo G., Trojano L., Maniscalco M. (2022). Cognitive Impairment in Convalescent COVID-19 Patients Undergoing Multidisciplinary Rehabilitation: The Association with the Clinical and Functional Status. Healthcare.

[48] di Pietro D.A., Comini L., Gazzi L., Luisa A., Vitacca M. (2021). Neuropsychological Pattern in a Series of Post-Acute COVID-19 Patients in a Rehabilitation Unit: Retrospective Analysis and Correlation with Functional Outcomes. Int. J. Environ. Res. Public Health.

[49] Pistarini C., Fiabane E., Houdayer E., Vassallo C., Manera M.R., Alemanno F. (2021). Cognitive and Emotional Disturbances Due to COVID-19: An Exploratory Study in the Rehabilitation Setting. Front. Neurol..

[50] Paneroni M., Vogiatzis I., Bertacchini L., Simonelli C., Vitacca M. (2021). Predictors of Low Physical Function in Patients With COVID-19 With Acute Respiratory Failure Admitted to a Subacute Unit. Arch. Phys. Med. Rehabil..

[51] Ortelli P., Ferrazzoli D., Sebastianelli L., Engl M., Romanello R., Nardone R., Bonini I., Koch G., Saltuari L., Quartarone A. (2021). Neuropsychological and Neurophysiological Correlates of Fatigue in Post-Acute Patients with Neurological Manifestations of COVID-19: Insights into a Challenging Symptom. J. Neurol. Sci..

[52] Daroische R., Hemminghyth M.S., Eilertsen T.H., Breitve M.H., Chwiszczuk L.J. (2021). Cognitive Impairment After COVID-19—A Review on Objective Test Data. Front. Neurol..

[53] Hampshire A., Trender W., Chamberlain S.R., Jolly A.E., Grant J.E., Patrick F., Mazibuko N., Williams S.C., Barnby J.M., Hellyer P. (2021). Cognitive Deficits in People Who Have Recovered from COVID-19. EClinicalMedicine.

[54] Sasannejad C., Ely E.W., Lahiri S. (2019). Long-Term Cognitive Impairment after Acute Respiratory Distress Syndrome: A Review of Clinical Impact and Pathophysiological Mechanisms. Crit. Care.

[55] Hellgren L., Birberg Thornberg U., Samuelsson K., Levi R., Divanoglou A., Blystad I. (2021). Brain MRI and Neuropsychological Findings at Long-Term Follow-up after COVID-19 Hospitalisation: An Observational Cohort Study. BMJ Open.

[56] Wahlgren C., Divanoglou A., Larsson M., Nilsson E., Balkhedb Å.Ö., Niward K., Thornberg U.B., Gudmundsson E.L., Levi R. (2022). Rehabilitation Needs Following COVID-19: Five-Month Post-Discharge Clinical Follow-up of Individuals with Concerning Self-Reported Symptoms. EClinicalMedicine.

[57] Elizabeth Wilcox M., Brummel N.E., Archer K., Wesley Ely E., Jackson J.C., Hopkins R.O. (2013). Cognitive Dysfunction in ICU Patients: Risk Factors, Predictors, and Rehabilitation Interventions. Crit. Care Med..

[58] Blazhenets G., Schroeter N., Bormann T., Thurow J., Wagner D., Frings L., Weiller C., Meyer P.T., Dressing A., Hosp J.A. (2021). Slow but Evident Recovery from Neocortical Dysfunction and Cognitive Impairment in a Series of Chronic COVID-19 Patients. J. Nucl. Med..

[59] Zampogna E., Paneroni M., Belli S., Aliani M., Gandolfo A., Visca D., Bellanti M.T., Ambrosino N., Vitacca M. (2021). Pulmonary Rehabilitation in Patients Recovering from COVID-19. Respiration.

[60] Daynes E., Gerlis C., Chaplin E., Gardiner N., Singh S.J. (2021). Early Experiences of Rehabilitation for Individuals Post-COVID to Improve Fatigue, Breathlessness Exercise Capacity and Cognition–A Cohort Study. Chron. Respir. Dis..

[61] Patil M., Gupta A., Khanna M., Taly A.B., Soni A., Keshav Kumar J., Thennarasu K. (2017). Cognitive and Functional Outcomes Following Inpatient Rehabilitation in Patients with Acquired Brain Injury: A Prospective Follow-up Study. J. Neurosci. Rural. Pract..

[62] Deng J., Zhou F., Hou W., Silver Z., Wong C.Y., Chang O., Huang E., Zuo Q.K. (2021). The Prevalence of Depression, Anxiety, and Sleep Disturbances in COVID-19 Patients: A Meta-Analysis. Ann. N. Y. Acad. Sci..

[63] Shanbehzadeh S., Tavahomi M., Zanjari N., Ebrahimi-Takamjani I., Amiri-arimi S. (2021). Physical and Mental Health Complications Post-COVID-19: Scoping Review. J. Psychosom. Res..

[64] Matalon N., Dorman-Ilan S., Hasson-Ohayon I., Hertz-Palmor N., Shani S., Basel D., Gross R., Chen W., Abramovich A., Afek A. (2021). Trajectories of Post-Traumatic Stress Symptoms, Anxiety, and Depression in Hospitalized COVID-19 Patients: A One-Month Follow-Up. J. Psychosom. Res..

