# Supplementary material for: Cognitive Deficits among Individuals Admitted to a Post-Acute Pneumological Rehabilitation Unit in Southern Italy after COVID-19 Infection
Source: Brain Sci. 2023 Jan 1;13(1):84. doi: 10.3390/brainsci13010084 (PMC9857316; doi:10.3390/brainsci13010084)
Supplement: Supplementary file 1 [file brainsci-13-00084-s001.zip › brainsci-2082983-supplementary.pdf]

**Supplementary Materials**

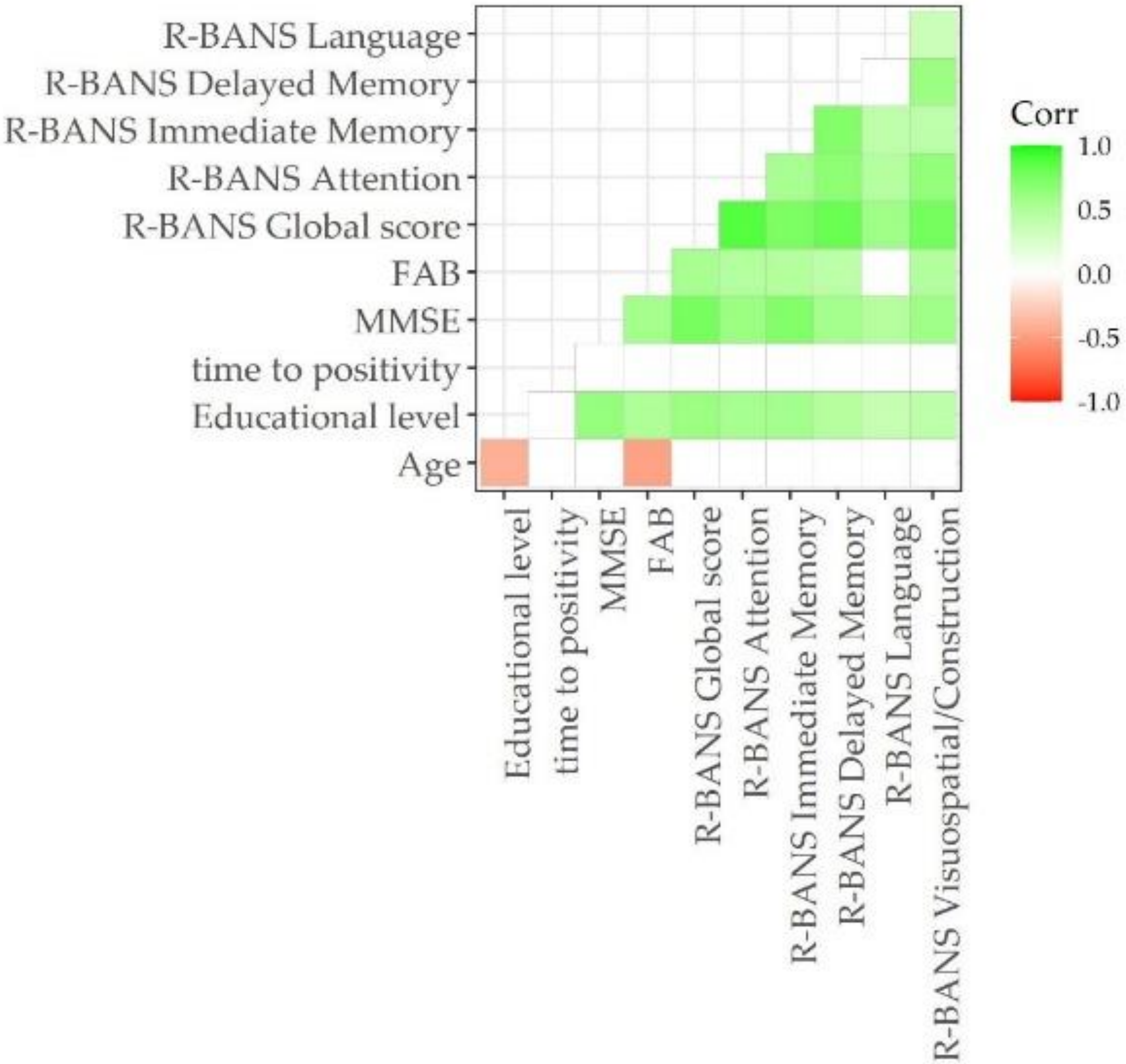


**Figure S1.** *Spearman non-parametric correlation tests used to investigate correlations between variables.*
